# p107 in the public eye: an Rb understudy and more

**DOI:** 10.1186/1747-1028-5-9

**Published:** 2010-04-02

**Authors:** Stacey E Wirt, Julien Sage

**Affiliations:** 1 Departments of Pediatrics and Genetics, Stanford Medical School, Stanford, CA 94305, USA; 2 Program in Cancer Biology, Stanford Medical School, Stanford, CA, 94305, USA

## Abstract

p107 and its related family members Rb and p130 are critical regulators of cellular proliferation and tumorigenesis. Due to the extent of functional overlap within the Rb family, it has been difficult to assess which functions are exclusive to individual members and which are shared. Like its family members, p107 can bind a variety of cellular proteins to affect the expression of many target genes during cell cycle progression. Unlike Rb and p130, p107 is most highly expressed during the G1 to S phase transition of the cell cycle in actively dividing cells and accumulating evidence suggests a role for p107 during DNA replication. The specific roles for p107 during differentiation and development are less clear, although emerging studies suggest that it can cooperate with other Rb family members to control differentiation in multiple cell lineages. As a tumor suppressor, p107 is not as potent as Rb, yet studies in knockout mice have revealed some tumor suppressor functions in mice, depending on the context. In this review, we identify the unique and overlapping functions of p107 during the cell cycle, differentiation, and tumorigenesis.

## Review

### Introduction

The *Rb *tumor suppressor was first identified as the gene whose loss causes hereditary retinoblastoma in children [1-4]. Further studies identified a variety of cancers with mutations in the *Rb *gene or deregulation of the Rb pathway, leading to the hypothesis that Rb is a major tumor suppressor whose loss of function is a common factor in most human tumors [5]. Independent studies with viral oncogenes such as SV40 Large T antigen, adenovirus E1A, and human papilloma virus E7 showed that Rb could be bound and inactivated by these oncoproteins, leading to the transformation of various cell types [6-11]. These discoveries have paved the way for over 20 years of studies on the mechanisms of cell cycle control and tumor suppression. But Rb was not the only protein that could bind to these viral oncoproteins, and the exact regions necessary for binding to Rb could also bind two other cellular proteins, eventually identified as p107 and p130 [11-13]. Together, the *Rb *gene family makes up a critical component of the cell cycle machinery and is conserved across many species [Reviewed in [14]]. However, we still do not have the answers to many essential questions about how these genes function and in what cellular context they are required for cell cycle control and tumor suppression. Additionally, the overlapping functions of each of the three genes further complicates our understanding of how they control critical cellular functions such as exit from and entry into the cell cycle, differentiation, and cell death. We will focus this review on our understanding of p107 and what is known about its functions in the cell cycle, cellular differentiation, and tumor suppression.

### Evolution of the Rb gene family

**Figure 1:**
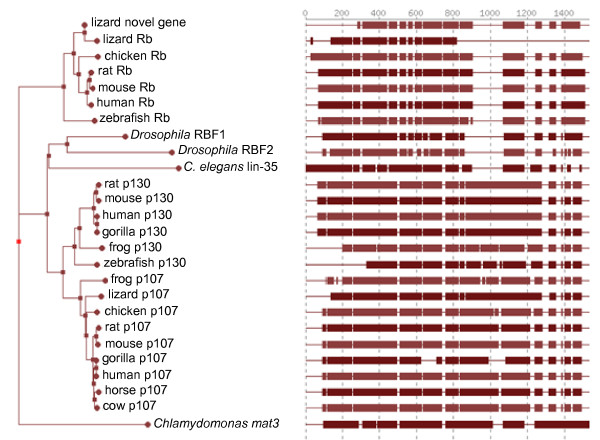
**Phylogenetic tree illustrating the evolutionary relationships among *Rb *family homologs in several different species**. Branch length corresponds to the estimated evolutionary distance between protein sequences. The protein sequence homology is shown in blocks on the right. Tree was constructed using the Alignment Analyzer from the Sol Genomics Network [137]. These observations suggest that the ancestral *Rb *family gene was closer to *p107 *or *p130 *than to *Rb *itself.

*Rb*-related genes can be found across multiple species, including humans, mice, chickens, reptiles, flies, and even some plants. Most unicellular and lower organisms have only one *Rb*-related gene, while higher organisms tend to have two or three family members, perhaps reflecting an increasing complexity of cell cycle control in these species. For example, the unicellular alga *Chlamydomonas reinhardtii *only contains one *Rb*-like gene (*mat3*), whose loss leads to a deregulation of proliferation and a reduced cell size [15]. In yeast, the gene *Whi5 *appears to play a functionally similar role to Rb, despite a lack of sequence homology [16-18]. Most plant species seem to contain only one *Rb*-related gene, although recently a second *Rb*-related gene was identified in maize and rice [19,20]. *Caenorhabditis elegans *also contains one *Rb*-like gene, *lin-35*, which in sequence homology is more similar to *p107 *than to *Rb *[21]. Further up the evolutionary scale, an independent gene duplication is thought to have created two *Rb*-related genes in *Drosophila*, *RBF1 *and *RBF2 *[22-24]. Like *lin35 *in *C. elegans*, these two genes are more similar to mammalian *p107 *and *p130 *than they are to *Rb *itself. Interestingly, the gene duplication events in plants and flies are only two examples of many duplication events within the *Rb*-family over the course of evolution as additional duplication events have occurred in *Gallus gallus *(chicken), *Danio rerio *(zebrafish), and *Anolis carolinensis *(lizard) (Figure 1).

Based on the sequence similarities, many of the *Rb*-related genes in non-mammals resemble *p107 *or *p130 *more so than *Rb*, suggesting that *Rb *is likely to be the more recent addition to the family. In other words, *p107 *and *p130 *may be closer in sequence to the ancestral *Rb *gene than *Rb *itself. Regardless, it is striking that an ancestral *Rb*-like gene existed and has evolved across numerous species, often independently undergoing duplication multiple times throughout evolution. Furthermore, once duplicated, these *Rb*-related genes independently evolved complex regulatory systems in which one *Rb*-related gene can be transcriptionally regulated by the other, as was observed in flies, plants, and mammals [25-27]. This repeated and independent evolution of the gene family and its associated regulatory networks emphasize the critical role the *Rb*-related genes share in controlling the cell cycle across many different species. But why is there such a strong selection for multiple *Rb *genes across so many species? If two or more genes performed identical functions, then there would be no selection to keep all of them throughout evolution. One explanation for the selection to keep multiple Rb family members is that they have evolved unique functions in addition to their overlapping functions, which would allow for individual members to be essential in different cellular processes or cell types [28,29]. Another explanation is that they may individually become more specialized after duplication, with one gene losing some functions in favor of others [28,30]. Finally, it is also possible that the regulatory regions surrounding the Rb family genes become mutated instead of the coding sequence itself [30]. This type of mutation would allow the proteins to retain redundant functions, but be regulated in different ways or expressed in different patterns throughout the organism. Given the wide variety of ways in which the individual Rb family members are expressed and their unique and overlapping cellular functions in different organisms, it is likely that the Rb gene family underwent multiple rounds of subfunctionalization and neofunctionalization over time. These observations raise the question of why p107 was retained throughout the evolution of higher organisms, and what specific functions it performs in mammalian cells.

### Characterization and expression

**Figure 2:**
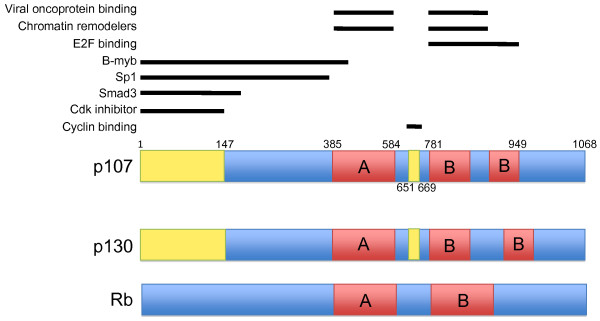
**Schematic diagram of the shared domains for p107, p130, and Rb**. p107 and p130 are more similar to each other than to Rb due to more extensive sequence homology and the shared Cyclin binding and Cdk inhibitory domains. Regions of p107 known to be important for specific protein interactions are shown above in blocked lines. The minimal sequences required for binding Sp1 and B-myb have not yet been identified, however the region N-terminal from the pocket domains has been shown to be critical for binding to both proteins.

p107, or Rb-like 1 (Rbl1) as it was originally named, was identified through its interaction with SV40 Large T antigen and adenovirus E1A [31]. Structurally, p107 contains a bipartite pocket structure similar to Rb, but it shares more sequence homology with p130. Both p107 and p130 contain the A and B regions of the pocket domain separated by a spacer region. Both p107 and p130 also contain insertions in the C-terminal B pocket that are absent from Rb, as well as a distinct Cyclin-binding domain in the spacer region between the A and B pocket domains. Additionally, p107 and p130 also contain a Cdk inhibitor domain in the N-terminus that is not present in Rb (Figure 2).

Unlike Rb and p130, p107 levels are generally, but not always, low in quiescent and differentiated cells and higher when cells proliferate [32-37]. p107 molecules can be detected in both the cytoplasm and the nucleus in various cell types. It is thought that p107 binds to repressor E2F family members such E2F4 in the cytoplasm to bring these repressors to their target genes in the nucleus [38,39]. During G1 and in early S phase of the cell cycle, high-resolution deconvolution microscopy has revealed that p107 and its family members can be found in perinuclear foci, where they co-localize with E2Fs and HDAC proteins [40,41]. These complexes are largely found in interchromatin regions, where active transcription is thought to occur. Whereas p130/E2F4 complexes are mainly found in G0 and G1 phase, p107/E2F4 complexes increase after G1 and are largely found in S phase [41]. These data suggest that p107 can recruit transcription factors from the cytoplasm and the nucleus to regulate transcription at the promoters of actively transcribed genes.

Of the three Rb family members, p107 is thought to be the most heavily regulated at the transcriptional level [26,36]. p107 itself is a known E2F target gene, containing two E2F consensus sites in its promoter [27]. Upon Rb ablation, increased E2F activity is thought to cause an increase in p107 expression, which may be able to compensate for the loss of Rb in certain contexts [42-44]. Indeed, many cell types express increased levels of p107 in the absence of Rb [45-49]. One hypothesis is that Rb directly controls p107 expression through direct binding to E2Fs at the p107 promoter. Interestingly, in the absence of p107, no significant increase is seen in Rb or p130 [50], suggesting that one of the main functions of p107 may be to serve as a backup for loss of Rb.

### Cell cycle functions

p107, like p130 and Rb, is a substrate for Cyclin/Cdk kinase activity during cell cycle progression, which is thought to cause p107 to release E2F transcription factors and relieve repression of target gene promoters [51-54]. In quiescent cells, p107 is usually expressed at low levels and is generally hypophosphorylated similarly to Rb; p107 becomes phosphorylated as cells progress towards S phase, with the first phosphorylation events occurring roughly when Cyclin D/Cdk4 is activated [33,55]. Thus, the highest expression levels of p107 in S phase correlate to a time when the protein is functionally deactivated by phosphorylation. After S phase, p107 remains phosphorylated throughout the rest of the cell cycle until the next G1 phase, when it is rapidly dephosphorylated, presumably by phosphatase 2A, and can bind E2F target genes in late G1 and S phase before it is hyperphosphorylated again [56-59].

Once bound to E2Fs, Rb family members repress transcription through a variety of methods. The Rb family can directly recruit chromatin modifying enzymes such as histone deacetylase HDAC1 (Figure 2), which alters chromatin structure around the E2F site to repress transcription at E2F target genes [60-68]; alternatively, Rb family members may interfere with pre-initiation complex assembly at the promoters of E2F target genes [69]. While Rb mainly associates with E2F1, E2F2, E2F3, and E2F4 *in vivo*, p107 preferentially binds to E2F4 and E2F5 at the promoters of E2F target genes in cycling cells [70-77]. In quiescent cells, p107 levels are generally low, and not detected at target gene promoters [56]. In the absence of Rb, however, p107 may play a compensatory role and can be found in complex with E2F1-3 [70], and transcription of E2F targets can be regulated normally, at least in certain contexts [45]. Interestingly, in the combined absence of p107 and p130, some E2F target genes are deregulated such as those coding for E2F1, Cyclin A2, B-myb, DHFR, and Cdc2, and these targets are different than the ones deregulated in the absence of Rb [45]. This evidence suggests that Rb cannot compensate for the loss of p107 and p130 at certain promoters, and that certain E2F target genes rely on either Rb or p107/p130 for normal expression and regulation. While the basis for this specificity is unknown, it points to some unique functions of p107 and p130 that are distinct from Rb. A remaining question is whether p107 and p130 themselves share the same targets, or whether they each bind to a distinct set of E2F target genes *in vivo*. Future experiments should aim to identify more extensive sets of target genes bound by individual Rb family members in various cellular contexts. Experiments such as genome-wide chromatin immunoprecipitation followed by sequencing (ChIP-Seq) with antibodies specific to p107, p130, and Rb in different cell types or at different phases of the cell cycle may help to shed light on this question.

In addition to its role as a transcriptional repressor of E2F activity, p107 may also control entry into S phase by regulating the levels of the F-box protein Skp2 within a cycling cell. p107 can down-regulate Skp2 levels, causing the stabilization of the cell cycle inhibitor p27 [78]. Stabilized p27 can then bind and inhibit Cyclin E/Cdk2 complexes, which are essential for the progression into S phase [78,79].

Recent evidence further points to a unique role for p107 during S phase, in addition to its function in late G1. While SAOS-2 cells transiently transfected with physiologic levels of Rb arrest in G1, the same cells transfected with physiologic levels of p107 arrest in both G1 and S phase [80]. A small pool of under-phosphorylated p107 persists throughout S-phase, and this pool may interact with other cell cycle regulators such as Smad3 to repress transcription of cell cycle genes like *c-Myc *[77]. In response to DNA damaging agents such as UV irradiation or addition of cisplatin, p107 can be rapidly dephosphorylated in cells progressing though S phase [81,82], and the phosphatase responsible for the de-phosphorylation of p107 may be protein phosphatase 2A [59,82]. This increase in hypophosphorylated p107 in response to DNA damage is independent of p53 or p21 activity, as cells with a null mutation in either inhibitor can still dephosphorylate p107 after DNA damage [82]. This evidence suggests a model in which massive dephosphorylation of p107 in response to genotoxic stress can contribute to the DNA damage response by invoking cell cycle arrest, although the exact mechanisms for how p107 induces an S-phase arrest are still unknown.

p107 and p130 bind to and inhibit Cyclin E/Cdk2 and Cyclin A/Cdk2 kinases through a unique spacer region in between the A and B pockets that is not present in Rb (Figure 2) [52,74,83-89]. This region is phosphorylated when bound by Cyclin/Cdk complexes [88]. In addition to the spacer region, there is a domain in the amino-terminus of both pl07 and p130 that can inhibit Cyclin/Cdk kinase activity similarly to the Cyclin-dependant kinase inhibitors p21 and p27 [83,88,89]. p107/Cyclin/Cdk complexes can be found in two distinct populations within a cell: those that contain E2F4/DP complexes and those that do not [89]. Recent evidence suggests that Cyclin D1 itself can bind to the promoters of many genes [90], and it would be interesting to determine whether Cyclin D binding had any correlation to known p107 or E2F target genes.

Through its N-terminus region, p107 binds the transcription factor Sp1 and represses Sp1 transcriptional activation, and this interaction may be unique to p107 among Rb family members [91,92]. In transient transfection assays [91], p107 can repress Sp1 transcription activation, and endogenous Sp1/p107/E2F4 complexes have been identified at the promoter of the *Fgfr1 *gene in chick myoblasts [92]. Additionally, the N-terminal domain of p107 can bind to the transcription factor B-myb, which competes with binding of Cyclin/Cdk complexes and prevents their sequestration by p107 [93]. The N-terminus of p107 can also bind to Smad3 in response to TGFβ signaling, and in this context p107 serves as an adaptor that is required to bring both E2F4-5/DP complexes and Smad3 to the nucleus. Once in the nucleus, p107/E2F4-5/Smad3 complexes bind to the promoter of *c-Myc *and repress its transcription. This interaction can explain how TGFβ signaling is able to selectively repress *c-Myc *transcription upstream of Cyclin/Cdk inactivation, and it is a unique function of p107, as Rb and p130 are unable to bind to Smad3 [77]. Interestingly, p107 can also directly bind c-Myc through the pocket domain and prevent its transactivation in transient transfection assays [94,95]. These experiments provide additional evidence for the many ways in which p107 is able to inhibit cell cycle progression through multiple interactions with various transcription factors and other proteins in addition to E2F (Summarized in Table 1).

**Table 1:** Summary of functional differences between p107, Rb, and p130.

*Function*	p107	p130	Rb	References
Binds to Smad3	yes	no	no	[77]

Binds to Sp1	yes	no	no	[91]

Binds to c-Myc	yes	unknown	no	[94,95]

Binds to Cyclins	yes	yes	no	[51,83-86,89,140]

Regulates chrondrocyte development *in vivo*	yes	yes	no	[58,102-104,139]

Regulates neural precursor populations through FGF and Hes1 *in vivo*	yes	no	no	[105,108,112]

Regulates cerebellar granule cell survival	yes	no	yes	[113]

Tumor suppressor	weak	weak	yes	[44,49,122,123,129,131-133,141]

### In vivo phenotypes for loss of p107 function in mice

Proper development requires the tight integration of cell cycle control, differentiation signals, migration, and cell death. Interestingly, numerous studies have demonstrated that Rb can not only affect cell cycle arrest in multiple cell lineages, but it can also interact with tissue-specific differentiation factors to promote the transcription of differentiation genes [Reviewed in [96-98]]. Like Rb, p107 has also been implicated in the regulation of numerous cell types during development, however its specific functions in different cell types are much less well defined. Does p107 affect differentiation largely though its influence on cell cycle control? Or can it, like Rb, integrate control of classical cell cycle genes and tissue-specific differentiation genes?

*p107*-deficient mice in a mixed 129/Sv:C57/BL6 background are viable and fertile, and mouse embryonic fibroblasts (MEFs) derived from these animals display no significant cell cycle defects [50]. Interestingly, *p107*-deficient mice in a Balb/c background show a severe postnatal growth deficiency, as well as myeloid hyperplasia in the spleen and liver. MEFs and myoblasts derived from these animals exhibit increased proliferation that was associated with constitutive expression of Cyclin E [99]. These mice have a significant decrease in white adipose tissue differentiation, although this decrease in differentiation was shown to be due to the fact that *p107^-/- ^*pre-adipocytes could not upregulate Rb, which is required to initiate differentiation in vivo through interaction with *Pgc1α *[100].

p107 and p130 seem to play overlapping roles during embryonic development in the mouse, and one reason for the lack of severe phenotypes in the *p107^-/- ^*mouse may be due to compensation from p130 or Rb. *p107^-/-^;p130^-/- ^*embryos die at birth with multiple defects in tissue development. Interfollicular keratinocytes from *p107^-/-^;p130^-/- ^*newborns show impaired terminal differentiation in the epidermis, decreased numbers of hair follicles, and a developmental delay in hair, whisker, and tooth formation [101]. These defects may be due to a general decrease in the amount of critical signaling molecules such as BMP and p63 in the double knockout epidermis, and implies that p107 can contribute to epithelial development along with p130.

**Figure 3:**
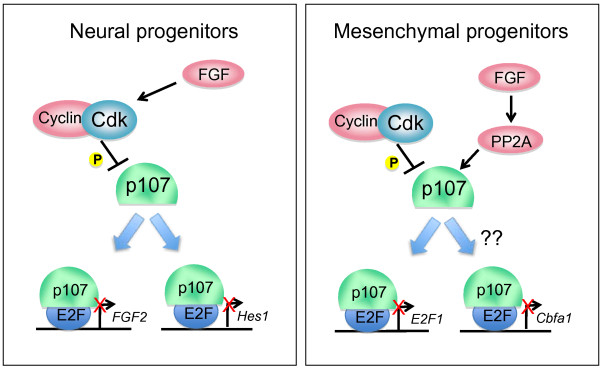
**Proposed mechanisms for how p107 can control neuronal differentiation and endochondral bone formation**. p107 can bind to E2Fs and potentially inhibit the transcription of *Hes1 *and *Fgf2*, two genes involved in cell cycle control, survival, and cell fate decisions during neurogenesis (left panel). During endochondral bone formation in mesenchymal progenitors, FGF signaling can induce the direct binding and de-phosphorylation is p107 by PP2A, which then leads to p107-mediated repression of target genes. Although the direct targets of p107 in this context have yet to be identified, candidates such as E2F1 and Cbfa1 have both shown to be critical mediators of bone and chondrocyte development and are deregulated in the absence of p107 and p130 [138,139].

p107 has also been implicated in the control of bone and cartilage development. Double knockout *p107^-/-^;p130^-/- ^*embryos as well as *p107^-/-^;p27^-/- ^*embryos display defects in ossification of the long bones and chondrocyte proliferation [102,103]. During chondrogenesis, FGF signaling induces a potent cell cycle arrest, and dephosphorylation of p107 is one of the earliest distinguishing events during this process, occurring 10-11 hours sooner than dephosphorylation of Rb and p130 [104]. Overexpression of Cyclin/Cdk complexes in developing chondrocytes prevented the dephosphorylation of p107 and completely abolished the growth suppression affects mediated by FGF signaling [58]. Biochemical studies in these cells have identified an interaction between the protein phosphatase PP2A and p107, suggesting a model in which FGF signaling stimulates PP2A to rapidly dephosphorylate p107, which results in a robust cell cycle arrest (Figure 3). Interestingly, FGF signaling in most other cells types has the opposite affect on cell growth; FGF signaling in these tissues triggers rapid phosphorylation of Rb family members and cell proliferation.

In the adult mouse brain, p107 expression is unique from the other Rb family members in that it is restricted to cycling progenitor cells in the ventricular zone, and its expression decreases as these cells begin to differentiate into cortical neurons [46,105]. Rb itself remains fairly consistently expressed throughout the transition from progenitor to neuron, and p130 expression increases with neuronal differentiation [106,107]. In the developing cortex of the mammalian brain, p107 may regulate the decision for a progenitor cell to exit the cell cycle and commit to a neuronal fate (Figure 3). p107 has been shown to negatively regulate neural precursor cell self-renewal both *in vitro *and *in vivo *[105]. Newly committed neurons that lack p107 die in the ventricular zone (VZ) before they can begin migrating out of the VZ or express markers associated with neuronal differentiation [108]. This apoptosis in the ventricular zone causes mice without p107 to have decreased numbers of neurons in the developing frontal cortex.

The mechanisms for how p107 can control cell cycle exit and fate decisions in neural progenitors are still relatively unknown. One potential explanation is that p107 acts through the repression of *Hes1*, a key downstream target of the Notch signaling pathway, since *p107*-deficient animals display increased Hes1 signaling (Figure 3). Several E2F binding sites have been located in the *Hes1 *promoter, and loss of one allele of *Hes1 *is enough to partially restore the numbers of neural progenitors to wild type levels in *p107^-/- ^*brains [108]. This interaction seems to be specific to p107, as Rb cannot repress the expression of the *Hes1 *promoter in luciferase assays. To date, however, *Hes1 *has yet to be proven to be a direct target of p107.

p107 may also be acting through the FGF growth factor signaling pathway to control the numbers of neural precursors in embryonic and adult brains (Figure 3). FGF is a critical factor to promote the proliferation and survival of neural precursors in the developing embryonic brain [109-111]. Like *Hes1*, the expression of *Fgf2 *(but not *Fgf1*, *Fgfr1*, or *Fgfr2*) is increased in neural progenitors of *p107*-deficient brains. Recently, p107 was shown to repress E2F3 activity at the promoter of *Fgf2*, an essential growth factor that modulates the population of neural precursors in the developing brain [112]. However, this model for p107 regulation of neural precursors does not explain why the increase in cell death observed in *p107^-/- ^*brains, as increased FGF2 would be expected to promote survival. Therefore, the mechanisms driving apoptosis in *p107^-/- ^*progenitors may be independent of FGF2 signaling, and have yet to be identified.

The two examples in chondrocytes and neurons suggest a scenario in which FGF signaling can indirectly activate p107, which could then repress FGF signaling through direct promoter binding. Although this feedback loop between p107 and FGF has yet to be identified within one specific cell type, it suggests the potential for a complex regulatory system for p107 and growth signaling during development.

Recent studies have uncovered functions of p107 that overlap with Rb during development. Additional loss of *p107 *shortened the lifespan of *Rb^-/- ^*embryos from birth to E14.5, and further increased the abnormal levels of proliferation and apoptosis that are present in the central nervous system and the ocular lens of *Rb*-deficient embryos. Mutation of *p107 *in an *Rb*-deficient background also caused heart development defects that were not seen in the *Rb*-deficient or *p107*-deficient embryos alone. These heart defects are likely the result of blood vessel endothelium and endocardial cell proliferation in the absence of both Rb and p107. Additionally, the cerebellar architecture is severely disrupted in adult mice lacking both Rb and p107 in the dorsal mid-hind brain junction, and these mice exhibit impaired terminal differentiation and migration defects in granule cell precursors, in addition to increased granule cell apoptosis upon maturation [113]. While the mechanisms underlying these defects are unknown, it is possible that p107 may be acting as a co-factor for neuron-specific proteins such as NeuroD1 [114], a basic helix-loop-helix transcription factor known to be important in the development of mature neurons [115]. Thus, p107 can partially or fully compensate for the loss of Rb in several tissues during development.

### Tumor suppression

*Rb *is mutated in a variety of sporadic and familiar human cancers, most notably in pediatric retinoblastoma and osteosarcoma. Mutations in *p107 *itself have not been observed in human tumors [116]. So far, the only observed deletion of p107 has been characterized in myeloproliferative disorders, where a large region of chromosome 20q containing up to 115 genes is deleted [116-118]. Despite the lack of mutation or deletion of *p107 *in human tumors, it still may play a role in tumorigenesis, as mutations in upstream regulators of the Rb family are common [119,120]. Inactivation of inhibitors such as p16, or activating mutations in Cyclin/Cdk complexes functionally inactivate Rb, p107, and p130 by hyperphosphorylation, suggesting that inactivation of all three Rb family members is necessary for tumorigenesis in multiple settings [116]. This finding highlights the ability of the Rb family proteins to fulfill overlapping or redundant roles in a variety of cellular contexts.

It is clear that p107 is not a strong tumor suppressor by itself, as mice with mutations in *p107 *do not develop spontaneous tumors [102]. Since p107 and p130 have overlapping functions during development, it was postulated that compound mutation of both genes might give rise to tumors in mice. Studies of heterozygous *p107^+/-^;p130^-/- ^*and *p107^-/-^;p130^+/- ^*mice revealed no spontaneous loss of either *p107 *or *p130 *allele and no obvious tumor phenotypes [121]. While cancer development in adult *p107^-/-^;p130^-/- ^*mice has not been described, these studies suggest that p107 and p130 do not by themselves have tumor suppressor functions in the mouse. However, these observations do not exclude the possibility that p107 and/or p130 may act as tumor suppressors in other contexts.

Interestingly, in the context of *Rb *loss, p107 can contribute some tumor suppressor functions within a cell (Table 2). Mice with mutations in *Rb *specifically in the epidermis develop epidermal hyperplasia and hyperkeratosis, however these mice do not develop tumors. Compound mutation of both *Rb *and *p107 *leads to papillomatous lesions within about 4 weeks; these lesions progress to squamous cell carcinomas shortly thereafter [122]. Just one wild-type allele of *p107 *is enough to confer tumor suppression in this tissue [122,123]. Interestingly, one allele of *p107 *is also sufficient to confer tumor suppression in studies of myeloproliferation in *Rb *family triple knock out mice [124,125]. The lung epithelium is also sensitive to *p107 *loss in the absence of *Rb*. *Rb^-/-^;p107^-/- ^*lungs show increased proliferation compared to *Rb^-/- ^*lungs, and double knockout lung epithelia develop adenomas or adenocarcinomas by 5 to 15 months of age. In contrast, *Rb^-/-^;p130^-/- ^*lungs did not develop spontaneous tumors in this setting, indicating that p107, but not p130, can enhance Rb tumor suppressor activity in the lung epithelium [126].

**Table 2:** Summary of *p107*-deficient mouse models and their phenotypes.

*Genotype*	Strategy	Lethality	Major Phenotypes	References
*p107^-/-^(Balb/c)*	Germline	Viable	Ectopic myeloid hyperplasia in the spleen and liver, severe postnatal growth deficiency, fibroblasts and myoblasts have increased cell cycle kinetics, decreased white adipose tissue.	[99,100]

*p107^-/-^(mixed)*	Germline	Viable	No gross abnormalities, expanded neural progenitor pool in the embryonic and adult brain.	[105,108]

*Rb^+/-^;p107^-/-^*	Germline	Viable	Pituitary tumors, reduced viability and growth retardation after birth until ~3 months of age, vaginal atresia (females).	[50]

*Rb^+/-^;p107^-/-^*	Chimera	Viable	Pituitary glad tumors, adenocarcinoma of the caecum, osteosarcoma, lymphosarcoma, occasional retinal dysplasia but no retinoblastoma.	[130]

*Rb^-/-^;p107^-/-^*	Chimera	Viable	Retinoblastoma development between 1 - 3 months of age, adult mice obtained at low frequency, apoptosis in the retina.	[130]

*Rb^-/-^;p107^-/-^*	Germline	Lethal E11.5	Accelerated apoptosis in the liver and CNS.	[97]

*Mox2Cre;Rb*^*lox*/*lox*^*;p107^-/-^*	Conditional (embryo)	Lethal E13.5-E14.5	Hyperproliferation of the CNS, lens, blood vessel endothelial cells. Double-outlet right ventricle (DORV) heart defect.	[142]

*p107^-/-^;p130^-/-^*	Germline	Birth	Hyperproliferation of chondrocytes, defective endochondral bone development, increased epidermal proliferation, decreased number of hair follicles, developmental delay in whisker, hair, and tooth formation.	[101,102]

*Rb^-/-^;p107^-/-^;hIRBPp53DD*	Chimera	Viable	Retinoblastoma.	[44]

*SPC-rtTA;tetCre;Rb*^*lox*/*lox*^*;p107^-/-^*	Conditional (lung)	Viable	~70% of mice develop lung adenoma or adenocarcinoma.	[126]

*K14Cre;Rb*^*lox*/*lox*^*;p107^-/-^*	Conditional (skin)	Viable	Spontaneous squamous cell carcinomas.	[49]

*NesCre;Rb*^*lox*/*lox*^*;p107^-/-^*	Conditional (retina)	Viable	Retinal dysplasia, high levels of apoptosis in the retina.	[129]

*Chx10Cre;Rb*^*lox*/*lox*^*;p107^-/-^*	Conditional (retina)	Viable	Unilateral retinoblastoma, 60% penetrant, delayed onset compared to *Rb/p53/p107*.	[132]

*Chx10Cre;Rb*^*lox*/*lox*^*;p53*^*lox*/*lox*^*; p107^-/-^*	Conditional (retina)	Viable	Aggressive bilateral retinoblastoma, 100% penetrant.	[132,141]

*Chx10Cre;Rb*^*lox*/*lox*^*;p130^-/-^;p107*^+/-^	Conditional (retina)	Viable	Differentiated horizontal neurons of the Inner Nuclear Layer re-enter the cell cycle and form metastatic retinoblastoma.	[133]

*Pax6α Cre;Rb*^*lox*/*lox*^*;p107^-/-^*	Conditional (retina)	Viable	Unilateral retinoblastoma, 60% penetrant, delayed onset compared to Rb/p130.	[131,143]

*En2Cre;Rb*^*lox*/*lox*^*;p107^-/-^*	Conditional (dorsal mid-hindbrain)	Viable	Ataxia between P15 and P20, disorganized cerebellar architecture, shrunken dendritic arborization, laminar defects, hyperproliferation of granule cell precursors, and granule cell death upon differentiation.	[113]

*p107^-/-^;p27*^*D*51/*D*51^	Germline	Viable	Chondrocyte hyperproliferation, defective chondrocyte maturation, defective endochondral bone formation.	[103]

*Hes1^-/-^;p107^-/-^*	Germline	Lethal E12.5	Embryonic lethality due to null Hes1 mutation, restoration of normal numbers of neural precursors in embryos and adults.	[108]

While mutations of *Rb *in human patients predispose them to retinoblastoma and osteosarcoma, mice with mutations in *Rb *develop an entirely different spectrum of tumors; pituitary and thyroid tumors are the most common malignancies, but not retinoblastoma or osteosarcoma. This unexpected tumor spectrum in *Rb*-deficient mice may be due to a functional compensation by p107 or p130. Indeed, some evidence exists for an upregulation of p107 protein in the absence of Rb in murine retinas, as discussed above [127]. A critical question, however, is whether p107 and p130 can suppress tumorigenesis similarly to Rb, or whether they have different tumor suppressor capabilities altogether. p107 is expressed highly in retinal progenitors as they actively cycle during the late-stages of embryonic development. p130, on the other hand, is only expressed at later postnatal stages of development in post-mitotic neurons [45,127,128]. Consistent with this observation, the combined loss of *Rb *and *p107 *during embryogenesis resulted in massive retinal dysplasia, whereas compound deletion of *Rb *and *p130 *had the same affect as deleting *Rb *alone [129]. Massive retinal dysplasia is also seen in adult chimeric mouse models lacking both *Rb *and *p107 *in the retina [129,130]. These results pointed to a potential role for p107 in suppressing retinoblastoma in mice. The use of the Cre-lox technology and conditional mouse models revealed further insights into the tumor suppressor functions of p107. Retina-specific deletion of *Rb *on a *p107^-/- ^*background with *Pax6α -Cre *or *Chx10-Cre *mice leads to predominantly unilateral retinoblastomas with about 60% penetrance. Interestingly, *Rb^-/-^;p130^-/- ^*retinas in the same system develop bilateral tumors with half of the tumor latency [131,132]. The slower kinetics and partial penetrance of the *Rb^-/-^;p107^-/- ^*retinas suggests that p107 mutation in this context is not always sufficient for tumorigenesis. A critical question that remains from these studies is whether the *Rb^-/-^;p107^-/- ^*tumors still retain functional p130 [131]. Interestingly, mouse retinas with triple compound mutation of *Rb*, *p107*, and *p53 *develop much more aggressive bilateral retinoblastoma in only a few months [44], suggesting that indeed, additional mutations are necessary for retinoblastoma formation in this context.

While it appears that p130 is a more potent tumor suppressor than p107 in retinal progenitors, p107 can still function as a tumor suppressor in specific cell types in the mouse retina. Studies of post-mitotic differentiated neurons of the inner nuclear layer (INL) of the retina showed that Rb expression was redundant with p130 [127]. In the absence of both *Rb *and *p130 *in this cell type, presence of p107 was sufficient to prevent retinoblastoma. However, in the absence of even one copy in this context, *p107 *was shown to be haploinsufficient for retinoblastoma development. Aggressive retinoblastomas arise from *Chx10-Cre;Rb*^*lox*/*lox*^*; p130^-/-^;p107^+/- ^*horizontal neurons several weeks faster than retinoblastomas from *Chx10-Cre; Rb*^*lox*/*lox*^*; p130^-/- ^*retinas [133]. Thus, it appears that in the INL of the retina, one copy of *p107 *is not enough to prevent tumorigenesis, whereas in other contexts, such as in hematopoietic progenitors, one copy of *p107 *is strong enough to prevent tumorigenesis [124]. The mechanisms underlying these contextual differences have yet to be identified.

*Rb*-heterozygosity results in retinoblastoma with 100% penetrance in humans but is not sufficient to cause retinoblastoma in mice. This difference in mice may be due to the ability for other family members, namely p107, to compensate for the loss of Rb in this context [127]. Interestingly, human retinal cells do not upregulate *p107 *in response to Rb loss, whereas mouse retinal cells do [127]. This difference may be due to different transcriptional regulation of p107 expression in mouse and human retinas; both mouse and human *p107 *promoters contain two tandem E2F binding sites. The mouse promoter has a single point mutation in the proximal 3' E2F site that may affect the binding of E2Fs or other transcriptional machinery that is recruited there [27]. Alternatively, differences in the surrounding promoter regions may be able to explain why one species can upregulate *p107 *in response to Rb loss in specific contexts, while the other cannot. These differences in *p107 *transcriptional regulation may also be seen within the different tissues of the same organism. For example, if deregulation of the Rb pathway can be found in almost all human tumors, why, then, do *Rb*-heterozygous patients primarily only develop a narrow spectrum of tumors, mainly retinoblastoma and osteosarcomas? This discrepancy may be explained by the fact that some tissues can upregulate p107 in response to Rb loss, whereas others cannot. Indeed upregulation of p107 is seen in several cell types upon Rb loss, including mouse retinal progenitors [127], keratinocytes [48], hepatocytes [36], and lung epithelial cells [123,126]. Interestingly, p107 seems to be the main Rb family member that can perform this function, as upregulation of *p130 *is generally not seen in response to Rb or p107 or both [127].

## Conclusions

To fully understand the tumor suppressor functions of Rb in human tumors, it is important to understand the functions of each of the family members, both individually and as a group. In particular, p107 functions in cell cycle control and tumor suppression have remained elusive.

During cell cycle progression, p107 function may be divided into two categories; those that require E2F and those that do not. ChIP-Seq for Rb family members and E2F family members in normal cells would shed light on the normal binding patterns of these proteins and may identify promoters that are regulated by individual Rb family members or by several at once. Expanding upon this, one could then compare the binding profiles of the Rb family members in normal cells to that of tumor cells, cells in different phases of the cell cycle, or cells from different tissues. p107 can also interact with several other key transcription factors such as Sp1, B-myb, c-myc, and Smad3. The significance of these interactions is not well understood, and several questions remain. For example, is p107's ability to regulate the cell cycle mainly exerted through E2F repression or can the interaction with other transcription factors also arrest the cell cycle independently of E2Fα One way to answer these questions would be to take advantage of the fact that E2Fs bind different regions of p107 than do the other transcription factors.

Evidence for p107 function during S phase suggests that it may play a critical role outside of the control of G1. Rb and p130 have both been implicated in the control of G0 and G2 [134-136], so in some ways it is not surprising that p107 would also play a role outside of G1. However, evidence for an Rb family role during S phase has so far been scarce. It is striking that the highest protein levels of p107 are seen in S phase, a time when p107 should largely be inactivated by phosphorylation. What would be the advantage to having large pools of p107 sequestered within a cell after the transition into S phase? One hypothesis would be that in response to genotoxic stress, p107 would be rapidly dephosphorylated by PP2A and serve as a reservoir of a potent transcriptional repressor. Large pools of p107 may also be able to stabilize p27, which could inhibit the activity of the Cyclin/Cdk complexes. The identification of downstream targets of p107 in this context will shed light on the exact mechanism for this S-phase arrest.

It has yet to be determined whether p107 can serve as a tumor suppressor in the context of other mutations outside of the Rb pathway. To date, there have been no studies crossing mice with *p107 *mutations to mice carrying mutations in other known cancer causing genes outside of the Rb pathway. Furthermore, no studies have examined whether *p107 *mutation and DNA damaging agents can contribute to faster or more aggressive tumors. Future studies should clarify the specific contexts in which p107 can act as a tumor suppressor, with or without compound mutation of Rb.

Although much has been uncovered since the discovery of the Rb family, the complexities in functional overlap, regulation, and tumor suppressor abilities of each of the Rb family members is only just beginning to be explored. The use of transgenic, knock-in, and knock-out mouse studies, as well as in vitro cell culture systems will be critical to increase our understanding of the role of these genes during multiple cellular functions, and these techniques will continue to reveal the subtle and distinct ways in which these proteins can interact with each other as well as the hundreds of other proteins known to associate with them. More analysis of how the Rb family normally functions is needed to understand their functions within a single cell, in addition to their tumor suppressor capabilities.

## List of abbreviations

Rb: Retinoblastoma; Cdk: Cyclin-dependent kinase; HDAC: histone deacetylase; DHFR: dihydrofolate reductase; BMP: bone morphogenetic protein; FGF: fibroblast growth factor; VZ: ventricular zone; ChIP: chromatin immunoprecipitation.

## Competing interests

The authors declare that they have no competing interests.

## Authors' contributions

SW and JS designed and wrote the manuscript together. Both authors have read and approved the final manuscript.
